# Effect of C content on the microstructure and properties of in-situ synthesized TiC particles reinforced Ti composites

**DOI:** 10.1038/s41598-023-49783-x

**Published:** 2023-12-14

**Authors:** Ning Wang, Yongbum Choi, Kazuhiro Matsugi

**Affiliations:** 1 Department of Mechanical Science and Engineering, Graduate School of Engineering, Hiroshima University, 1-4-1 Kagamiyama, Higashi-Hiroshimasi, Hiroshimaken 739-8527 Japan; 2 Mechanical Engineering Program, Graduate School of Advanced Science and Engineering, Hiroshima University, 1-4-1 Kagamiyama, Higashi-Hiroshima, Hiroshimaken 739-8527 Japan

**Keywords:** Structural materials, Mechanical engineering

## Abstract

Titanium matrix composites (TMCs) have garnered substantial attention from researchers owing to their outstanding properties. Nonetheless, the strength and ductility of TMCs hardly co-exist and often show a trade-off between each other. In this study, we employ an ultra-thin graphite powder sheet as the carbon source and employ Ti/C composites with varying carbon contents, prepared via a layer-stacked laminated sintering method, to ensure a comprehensive in-situ reaction and uniform reinforcement distribution. With increasing carbon content, noticeable alterations occur in the size, concentration, and morphology of the titanium carbide (TiC) particles. The increase of TiC particle content is found to boost the ultimate tensile strength of the composite. However, this improvement comes at the expense of reduced elongation. Notably, as the carbon content reaches 1.81 wt%, the yield strength and ultimate tensile strength of the composites soar to 354.4 MPa and 575.4 MPa, respectively. These values represent a remarkable increase of 75.4% and 65.0% compared to pure titanium, while maintaining an acceptable elongation of 6.45%. This study unveils a promising approach for significantly enhancing the mechanical properties of titanium alloys.

## Introduction

TMCs have received a lot of attention due to their application prospects in various industrial fields such as automotive, aerospace, biomedical, and military industries. These composites are known for their exceptional specific strength, excellent corrosion resistance and biocompatibility^1–6^. However, in today's rapid industrial development, traditional titanium alloys are difficult to meet the increasing demand for high-performance materials in rapid industrialization^3–7^. TiC, renowned for its high hardness, wear resistance, and corrosion resistance, is widely regarded as an ideal reinforcement material for ceramics due to its density and coefficient of thermal expansion closely resembling those of titanium and titanium alloys^7–9^. In situ synthesis of TiC reinforced TMCs involves exothermic chemical reactions with titanium groups through additives, which is considered to be one of the most promising processes because it not only improves the performance of the TiC reinforcement material and reduces the cost, but it combines the high strength of the reinforcement with the high plasticity of the titanium matrix^10,11^. The mechanical properties of TMCs mainly depend on the composition and microstructure of the matrix and particles. Ensuring the optimum strength and stiffness of these composites depends on precise control of the interface^12^. Previous studies have shown that uneven distribution of ceramic reinforcement in the metal matrix can lead to unsatisfactory mechanical properties^13^. Traditional composite preparation techniques, including powder metallurgy and combustion-assisted casting, often rely on powdered titanium as a raw material or necessitate extremely high temperatures to melt the matrix, significantly limiting large-scale composite production^13,14^. In consideration of energy consumption and sustainability, it is imperative to develop a cost-effective TMC preparation process that can further expand the application potential of these composites. Spark plasma sintering (SPS) is another technique suitable for the preparation of TMCs and has unique advantages. However, there are currently limited data on the effects of SPS temperature on the microstructure and properties of graphite powder sheet and its related materials reinforced ti matrix composites. From the point of view of the formation of TiC in the heating process of titanium and carbon sources, SPS is an ideal choice for the preparation of Ti-TiC layered composites using titanium foils and different carbon sources^15,16^. It is known that sintering temperature and pressure have a significant effect on the densification behavior, microstructure evolution and property enhancement of matrix composites^17^.

In this study, our primary objective is to ensure a comprehensive in-situ reaction and uniform distribution of reinforcement within Ti/C composites containing varying carbon contents. We prepared an ultra-thin graphite paper as the carbon source through a simple method, blending polyvinyl alcohol (PVA) and graphite powder, and controlled the carbon content by adjusting the amount of graphite powder. Utilizing 0.05 mm thick titanium foil as the titanium matrix, we employed a layer-stacking sintering method to fabricate TiC-reinforced TMCs. This approach avoided the use of powders and the high temperatures required for metal melting, thereby reducing costs. Additionally, the effects of carbon content on the morphology of the reinforcement, the evolution of the microstructure of the matrix and the mechanical properties of the composites were systematically investigated through composition design and process optimization.

## Experiment

### Preparation of graphite powder sheets

**Figure 1:**
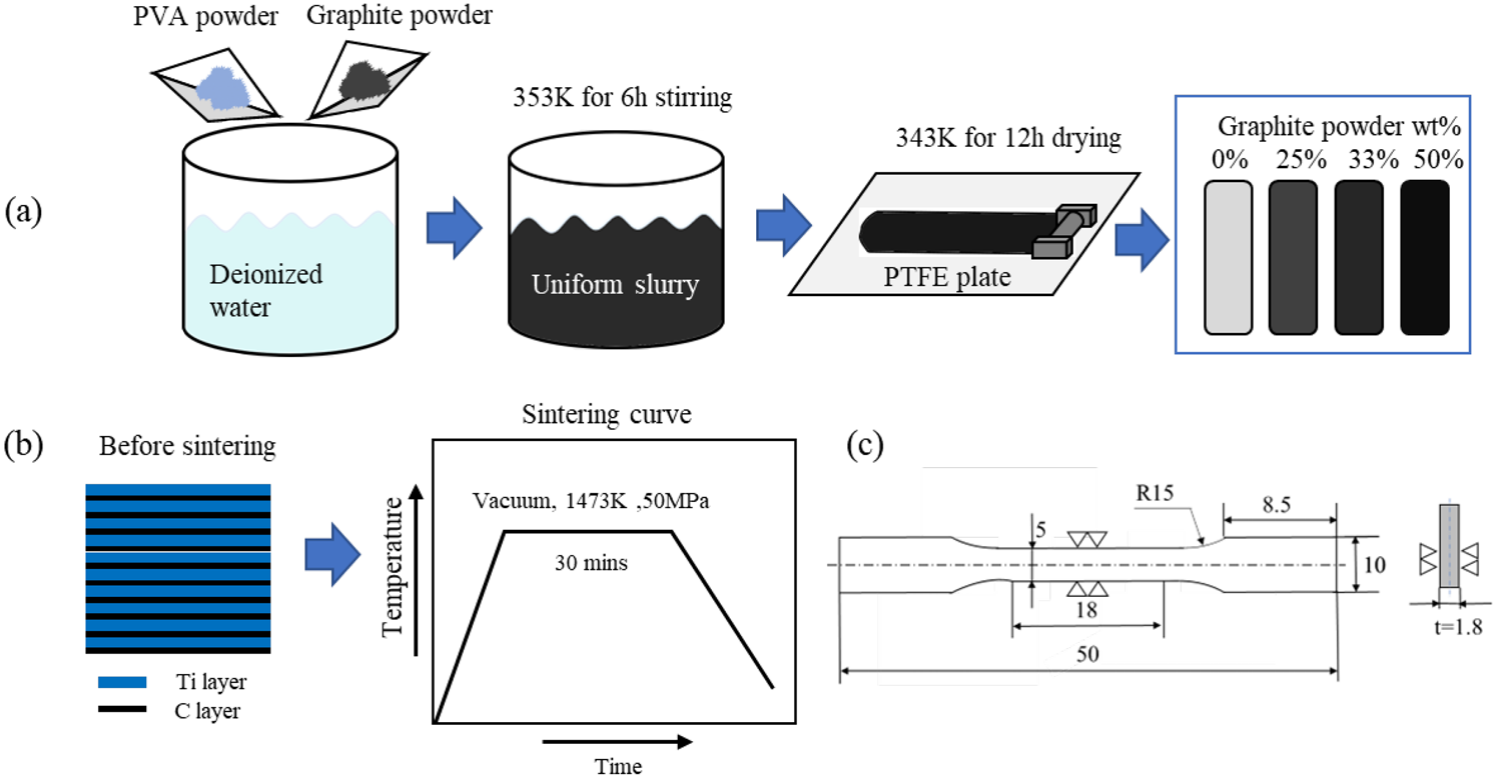
(**a**) Schematic illustration of fabricating processes of graphite powder sheet; (**b**) sintering process of TiC/Ti composites; (**c**) size of plate tensile specimen.

Graphite powder sheets were prepared by mixing PVA powder as a binder and graphite powder in various ratios (3:1, 2:1, and 1:1) in a glass container containing deionized water (11.0 vol.% PVA solution). The mixture was stirred using a magnetic stirrer for 6 h at 358 K to achieve a homogeneous slurry, as shown in Fig. 1a. This slurry was evenly applied to PTFE sheets and then placed in a drying oven at 338 K for 12 h to evaporate the solvent, resulting in the formation of graphite powder sheets. For comparison, sheets consisting solely of pure PVA were prepared using the same solution-evaporation method.

### Fabrication of TiC/Ti composites

Commercial purity Ti foils (99.5%, thickness of 50 µm) and graphite powder sheets were cut into ϕ10 mm disks and then cleaned in an ultrasonic bath of ethanol for 20 min. The Ti foils and graphite powder sheets were alternately stacked and sintered in a vacuum SPS furnace, as shown schematically in Fig. 1b. For convenience, the fabricated composites with PVA sheets and graphite powder sheets were labeled as TMC-P, TMC-1, TMC-2, and TMC-3, corresponding to the content of graphite powder. It is noteworthy that PVA leaves carbon residues after high-temperature pyrolysis, following the removal of volatile gases. In this study, the PVA used had a 4% carbon residue after high-temperature pyrolysis under argon atmosphere, a result consistent with literature reports. Therefore, the actual carbon content includes the amount of added graphite powder and the carbon generated after pyrolyzing PVA. The carbon contents of the samples were calculated to be 0.18, 1.27, 1.81, and 3.91 wt%, respectively.

### Characterization of TiC/Ti composites

The fabricated composites were mechanically ground and polished perpendicular to the lamellas. Phase structures were characterized using an X-ray diffractometer (XRD, Rigaku, MiniFlex600) with Cu-Kα radiation. Microstructures were examined using a scanning electron microscope (SEM, JEOL, JXA-8900RL, Japan) equipped with a backscattered electron (BSE) detector. Semi-quantitative compositional analysis was conducted using energy-dispersive X-ray spectrometry (EDS) attached to the Zeiss microscope.

### Mechanical properties measurement

Tensile strength and elongation percentage were determined for the samples using a Shimadzu AG-IS Tensile Tester and Trapezium-2 software in accordance with the ASTM test method E8M-11 standard, with a crosshead speed of 0.5 mm/min. The schematic illustration of specimen extraction and the dimensions of the tensile samples are depicted in Fig. 1c. Plate tensile specimens were polished before testing, and three specimens were tested for each set of measurements. Tensile fracture surfaces and subsurface morphologies were imaged using SEM.

## Results

**Figure 2:**
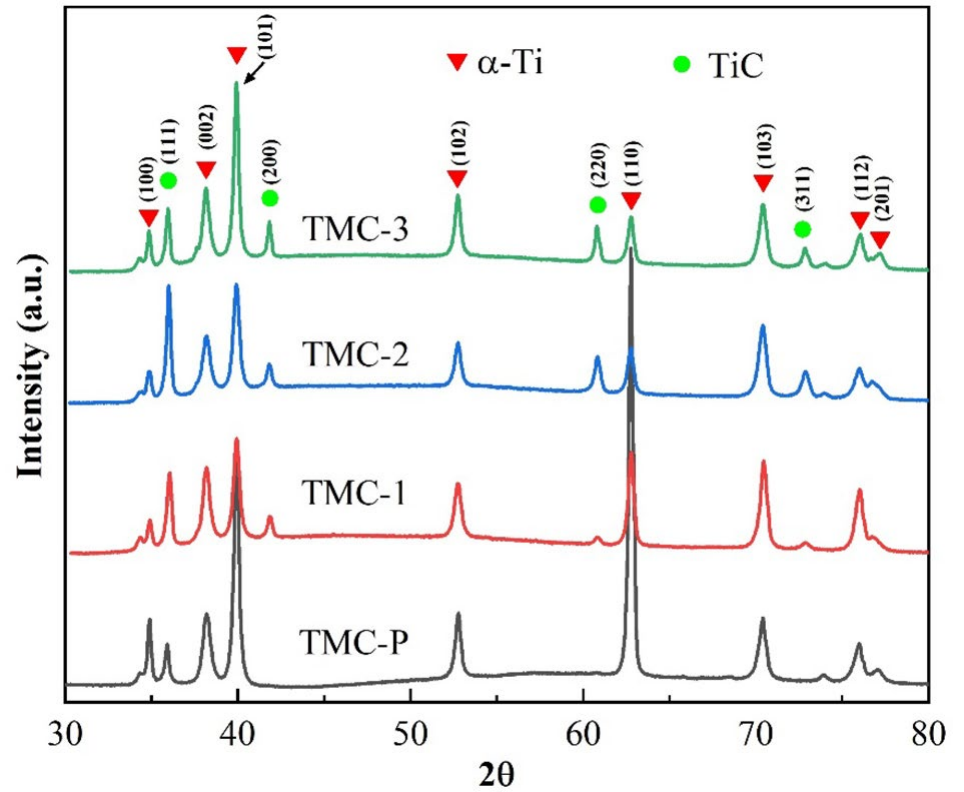
X-ray diffraction pattern of deposited samples with different C content.

Figure 2 displays the X-ray diffraction (XRD) results of cross-sectional views of TiC/Ti composites with varying carbon contents. These results reveal that the composites consist of two phases: TiC and α-Ti. In the case of sample TMC-P, only the diffraction peak corresponding to the (200) crystal plane of TiC is observed, primarily due to its low carbon content. This XRD analysis proves that TMCs reinforced with TiC can be fabricated by in situ technique utilizing the reaction between Ti and C.

**Figure 3:**
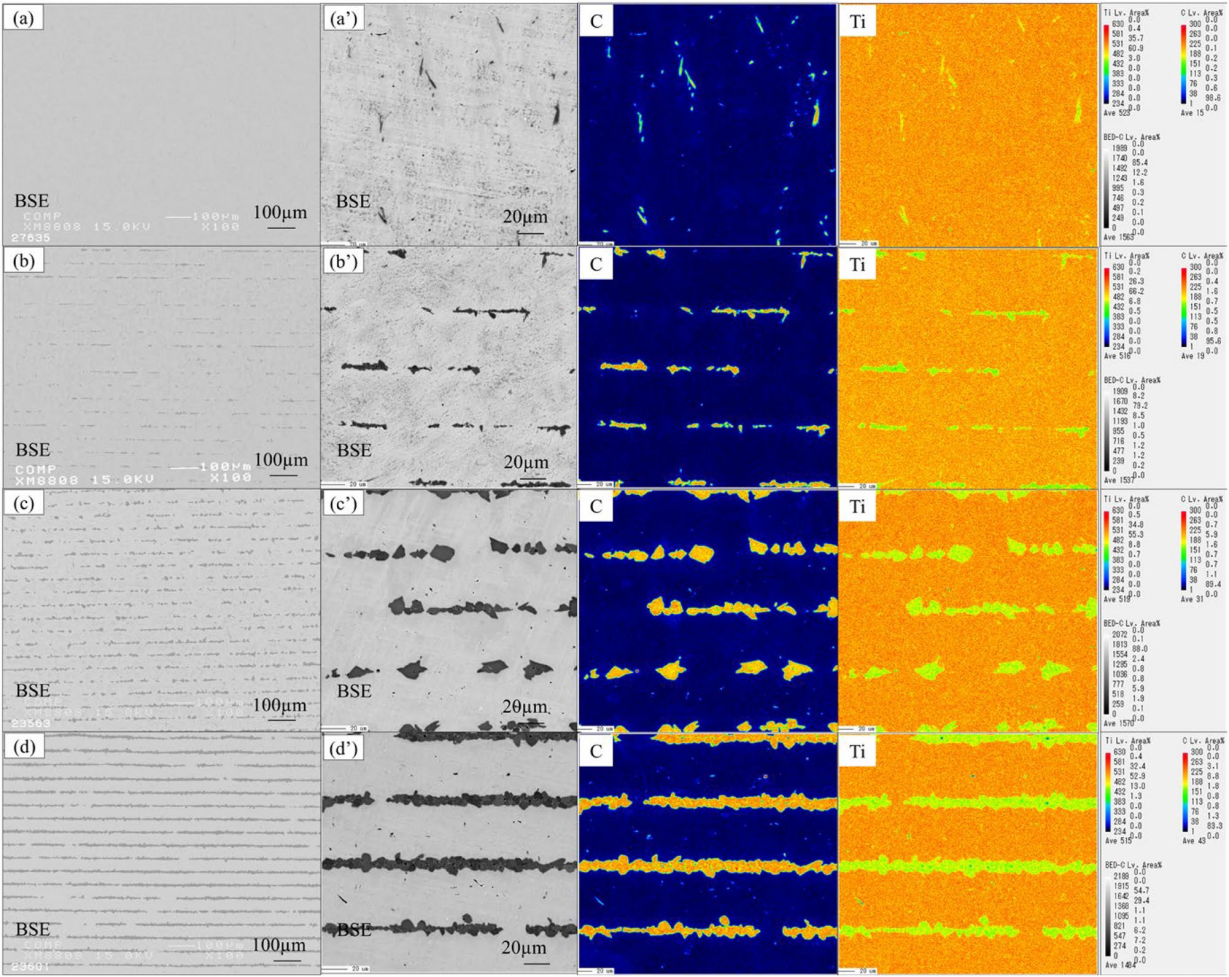
(**a**) Magnificat BSE images of TiC/Ti composites with different C content: (**a**) 0.18 wt% of TMC-P; (**b**) 1.27 wt% of TMC-1; (**c**) 1.81 wt% of TMC-2; (**d**) 3.91 wt% of TMC-3; (**a**’)–(**d**’) corresponding magnified mapping images.

Figure 3 shows the microstructures of sintered TiC/Ti composite materials with different carbon contents. It can be observed that all four composite materials prepared using the layer-stacking sintering method exhibit uniform microstructures. This even distribution of reinforcements helps prevent local strength deficiencies and concentrated stress, reducing the risk of brittle fracture under load. Additionally, it improves the material's strength by facilitating effective transfer and distribution of external loads^18^. For sample TMC-P, as shown in Fig. 3a, only a small number of gray particles and short rod-like eutectic TiC particles can be observed under magnification. From the high-magnification image, it can be observed that the particle size of the rod-like TiC is generally less than 5 μm. With an increase in carbon content to Fig. 3b, the short rod-like titanium carbides disappear, replaced by discontinuous TiC layers appear at original graphite powder sheet positions within the composite. TiC exhibits a granular morphology with sizes mainly in the range from 5 μm to 10 μm, and at these discontinuous locations, two layers of titanium foils bonded together. When the carbon content increases to Fig. 3c, due to the increased thickness of the graphite powder sheet, the formed TiC particles are relatively larger and can be clearly observed. The composite material still maintains a uniform distribution of TiC particles enhanced the morphology of the titanium matrix. As the carbon content increases to Fig. 3d, a multi-layered structure of TMCs becomes apparent. However, it can also be observed that in some areas, the multi-layered structure of TiC exhibits discontinuous morphologies, possibly due to the non-uniformity of the original graphite powder sheets.

**Figure 4:**
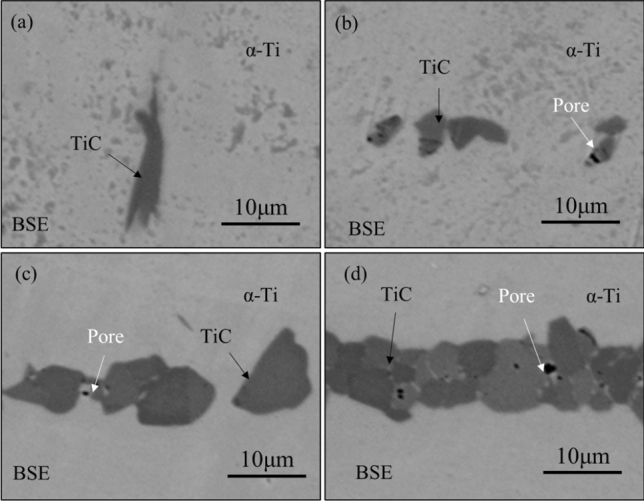
High magnification BSE images of TiC/Ti composites with different C content: (**a**) 0.18 wt% of TMC-P; (**b**) 1.27 wt% of TMC-1; (**c**) 1.81 wt% of TMC-2; (**d**) 3.91 wt% of TMC-3.

Figure 4 presents high-magnification images of titanium carbide particles in the matrix, providing a detailed view of their morphology. Figure 4a shows the morphology of two rod-shaped TiC particles connected, with no discernible pores observed at high magnification. As the carbon content increases, the morphology of titanium carbide primarily exhibits a particulate form with different size, either existing independently or aggregated together, uniformly distributed at the interface of the two titanium layers. Moreover, noticeable pores are evident in the TiC layer, the quantity of which increases with the elevated carbon content. The formation of these pores may be attributed to the inefficient expulsion of gases generated during the decomposition of PVA at high temperatures. Alternatively, it could be due to residual carbon. If the cause is indeed residual carbon, increasing the insulation duration may assist in its removal. However, further investigation is necessary to pinpoint the specific cause.

**Figure 5:**
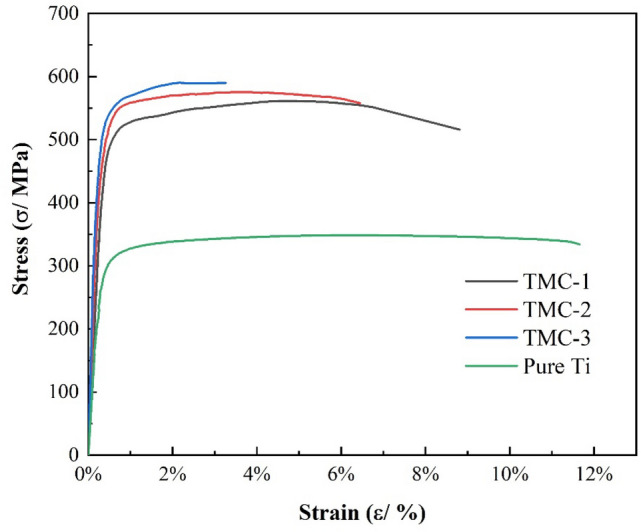
Room temperature tensile stress–strain curve of deposited TiC/Ti composites.

The room-temperature tensile results for TiC/Ti composites TMC-1, TMC-2, and TMC-3 are presented in Fig. 5. It can be observed that the incorporation of carbon significantly enhances the performance of pure titanium in the TiC-reinforced titanium-based composites prepared through the layer stacking in-situ synthesis method. However, this improvement comes at the cost of reduced ductility in the composites. The yield strength (YS) and ultimate tensile strength (UTS) of pure titanium are 202.0 MPa and 348.7 MPa, respectively. When the C content increases to 1.27 wt% and 1.81 wt%, the UTS increases to 561.4 MPa and 575.4 MPa, showing improvements of 60.1% and 65.0% respectively, compared to pure titanium. However, the corresponding elongation decreases by 24.4% and 44.7%, respectively. When the C content rises to 3.9 wt%, the composites exhibit a multi-layered microstructure and the UTS of the composites reach 590.2 MPa. In comparison to composites with dispersed granular TiC microstructures, there is no significant increase in tensile strength, but the elongation drastically drops to 3.26%, representing a decrease of 72.0%. The trend in the variation of tensile properties primarily arises from the lower C content, which results in smaller TiC particle diameters due to the thinner graphite powder sheet used. With an increase in C content, the diameter of TiC particles increases, and the particles exhibit distinct edges. Both of these samples share the morphology of discontinuous TiC particles uniformly distributed in the matrix. During tensile testing, the uniform dispersion of TiC particles significantly reduces the probability of localized stress concentration under external loading. This not only enables TiC particles to withstand higher stress during tensile testing but also reduces the rate of crack propagation. As the C content further increases, leading to a higher TiC content, the composite takes on a multi-layered structure. While the additional TiC particles enhance the strength of the composite, the clustering of TiC makes crack propagation within TiC more likely, leading to a drastic reduction in ductility.

**Figure 6:**
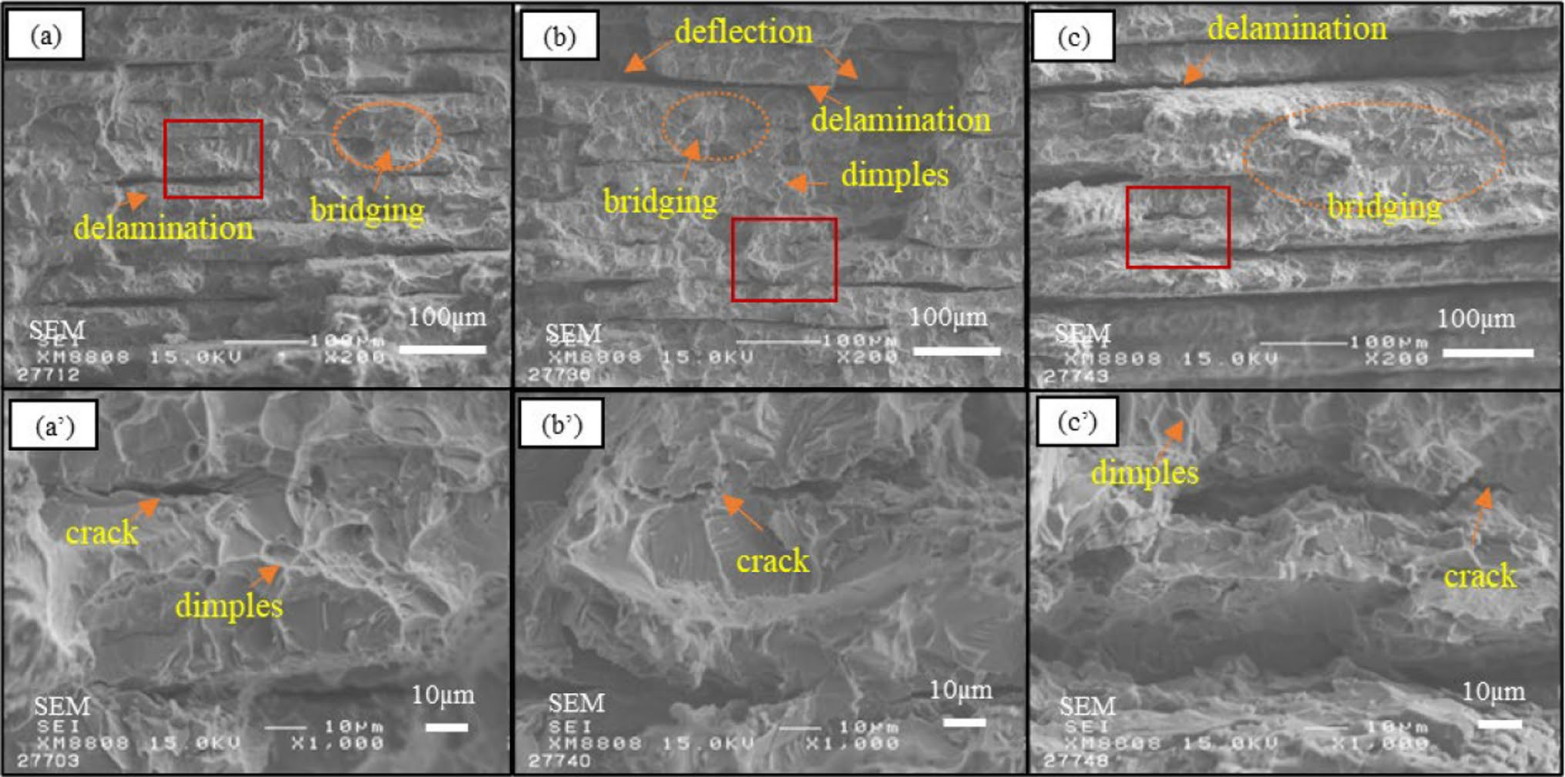
Scanning electron micrographs of tensile fracture surface of the titanium matrix composites tested with different C content: (**a**) 1.27 wt% of TMC-1; (**b**) 1.81 wt% of TMC-2; (**c**) 3.91 wt% of TMC-3.

To further investigate the deformation behavior of the matrix, the loading effects of TiC, and the fracture mechanisms in the failed composite materials, the fracture surfaces of the failed composites were conducted. Comparing the fracture surfaces of TMC-1 and TMC-2, no significant changes were found except for larger depressions and more delamination in the TMC-2 matrix. No evidence of particle pull-out features was observed on the fracture surfaces of all three samples, indicating a good interfacial system between the TiC particles and the matrix. Many dimples were observed on the fracture surface of the Ti layers, indicative of ductile fracture. The TiC layer between the two Ti layers exhibited delamination and cracks, attributed to the mismatch in strain between TiC and the matrix. This observation suggests that the applied load has transferred from the matrix to the TiC particles, facilitated by strong interfacial adhesion that provided effective load transfer, thereby enhancing the yield strength^8^. Additionally, some bridging was observed. As for TMC-3, more extensive delamination and continuous bridging were evident, indicating that TiC particles significantly embrittled the matrix, resulting in minimal plastic deformation. The bridging areas displayed crack surfaces, cleavage steps, and river patterns, signifying the coexistence of both brittle and ductile fractures (Fig. 6).

## Discussion

### Formation and morphological evolution of TiC

During the sintering process of composite materials, the principal mechanism responsible for the in-situ synthesis of TiC entails the diffusion of C atoms into the titanium matrix, leading to the subsequent formation of TiC. The feasibility of this reaction can be assessed through thermodynamic theory, which necessitates the computation of reaction formation enthalpy (Δ*H*) and Gibbs free energy (Δ*G*). As depicted below, under conditions where T < 1939K^19^:

1 $$\Delta H=-184571.8 +5.042T-(2.425 \times {10}^{-3} {T}^{2})-(1.958\times {10}^{6}/T)$$

2 $$\Delta G=-184571.8 +41.382T-5.042T lnT+2.425 \times {10}^{-3} {T}^{2}-9.79\times {10}^{5}/T$$

Calculated results reveal Δ*H* = –183.7 kJ/mol and Δ*G* = –173.2 kJ/mol. The Gibbs free energy of the reaction is negative at a temperature of 1473 K, indicating that the reaction can proceed spontaneously in thermodynamic range. Additionally, the formation enthalpy is also negative, representing the driving force behind the exothermic, self-sustaining, and rapid formation process of TiC.

TiC is a type of transition metal carbide distinguished by a face-centered cubic (FCC) NaCl-type structure, formed as smaller C atoms occupy octahedral positions, thus incorporating themselves into the titanium atomic lattice. Its actual composition typically deviates from stoichiometry and is symbolized as TiC_*x*_, where *x* signifies the ratio of C to Ti, with a range extending from 0.46 to 1.0^20,21^. Below 1153 K, the crystal structure of titanium adopts a hexagonal close-packed (HCP) configuration, with carbon atoms exhibiting solubility in titanium of approximately 0.05 wt%. Conversely, at temperatures surpassing 1153 K, titanium adopts a body-centered cubic (BCC) crystal structure, whereas the solubility of carbon atoms in titanium increases to around 0.15 wt%. These variations in carbon content influence the precipitation trajectory of TiC during the solidification process, ultimately shaping the morphology of TiC. In the TMC-P specimen, with a carbon content of 0.18 wt%, which is very close to 0.15 wt%, as the temperature reaches 1153 K, carbon atoms dissolve from the graphite layers, with some carbon atoms diffusing into the titanium matrix. As the temperature decreases below 1153 K, the reduced solubility prompts carbon atoms to precipitate, selectively diffusing along the (0001) _α-Ti_// (111) _TiC_ planes into the tetrahedral interstices, replacing Ti positions, ultimately resulting in the formation of equiaxed TiC particles in the form of short rods or equiaxed shapes^3,22^.

### Strengthening mechanisms

TiC particles exhibit exceptional mechanical properties, with an elastic modulus of 450 GPa and a microhardness of 3200 kg/mm^2^, significantly surpassing the mechanical performance of pure titanium (106.4 GPa and 150 kg/mm^2^)^8^. When the load was applied, the hard TiC particles can bear part of the load. Typically, the use of reinforcing materials to enhance the strength of TMCs is significantly influenced by the size, shape, proportion to the matrix, and the compatibility of the reinforcing material with the matrix. Additionally, the plastic deformation of the composite material is mainly affected by the matrix, and it is important to enhance the deformation resistance of the matrix, which is partly affected by the TiC content. For the strengthening factors of composite materials, it is generally believed that the strengthening effect of carbon mainly comes from two factors: one is the solid solution of C in the matrix, and the other is the bearing mechanism of TiC particles^23^. In the tensile tests conducted, the carbon content in all three samples exceeded the solubility limit in the titanium matrix. Furthermore, the increased matrix strength due to an elevated dislocation density constitutes a more pronounced strengthening effect.

**Figure 7:**
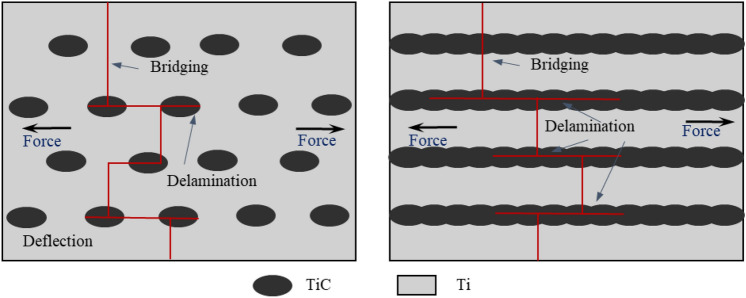
Schematic shown crack propagation mechanisms for two types of structure.

Figure 7 respectively illustrate schematics of the crack propagation mechanisms in discontinuous TiC particle-reinforced TMCs and multilayered TiC-reinforced TMCs. The crack propagation paths of the two structures are characterized by deflection and delamination, indicating that the ductile Ti layer can effectively release local stress and prevent crack propagation, thus improving the toughness of the composite. The Ti matrix between discontinuous TiC particles can accommodate more deformation, buffer the rapid expansion of cracks, and show better toughness. The fracture process of discontinuous TiC particle reinforced TMCs can be described by the fracture model proposed by Llorca and González^24^. At the initial stage of plastic deformation, with the matrix gradually strain hardening, the internal stress of the particles increases, resulting in the fracture of the critical reinforced particles. The load released by the broken particles is mainly carried by the surrounding matrix. With increasing strain, the fractured particles disperse uniformly throughout the entire sample. However, when the matrix's strain-hardening capacity saturates, as the matrix can no longer harden, the stress released by the fractured particles is transferred to the neighboring particles. The stress acting on the adjacent particles increases, causing more particle fractures. The ultimate fracture of the composite material is attributed to the ductile failure of the matrix, based on nucleation and void growth^25^.

As shown in Fig. 6, the fracture surface inspection confirmed the existence of TiC particle fracture, indicating that there is a strong interface adhesion between the in-situ synthesized TiC particles and the Ti matrix, thus ensuring the effective transfer of load and improving the yield strength. As the TiC particle fraction increases, the fracture surface becomes more uneven, resulting from the rapid extension of cracks. Notably, the particles in specimen TMC-2 are significantly larger than those in TMC-1. Once the TiC particle fraction is relatively high, especially with large particles and particle clusters in TiC, the cracks spread rapidly to the matrix due to the low plasticity of the matrix. Other studies have indicated that rapid coalescence of cracks occurs in large particles, leading to composite material fracture. Hence, reducing particle size may potentially improve the room-temperature tensile elongation of the composite material, which is worthy of further investigation. Furthermore, pores were observed on the surfaces of TiC in the discontinuous particle structure and in the composite materials with a multi-layered structure. These pores can also lead to premature failure. When microcracks form within the TiC crystals, they propagate along paths containing pores, accelerating the fracture of TiC crystals.

Additionally, an increase in the volume fraction of the reinforcement phase in multilayered TMCs results in an increase in dislocation density within the matrix, thereby improving yield stress. The fracture process in multilayered TMCs can be explained using an equivalent strain model based on laminated theory during uniaxial tension tests^26^. Because the elastic modulus of Ti and TiC is different, the internal stress is different under the action of external force. With the increase of load, the Ti layer has plastic deformation, and the brittle TiC layer has micro-cracks and expands, releasing the internal stress. However, the ductile Ti layer prevents further crack propagation. With the increasing of tensile stress, in order to release the constraints around the ductile Ti layer, extensive plastic deformation occurs in the Ti layer. At this stage, the Ti layer starts to bear the load until it eventually fractures^27^. Larger particles and particle clusters are more prone to premature fracture due to greater constraints on plastic flow in the matrix, which has been supported by previous studies^23^. Within the particle clusters, triaxial stress is applied to cause fracture between particles and within the matrix. The matrix inside the particle cluster is fractured, which leads to the propagation of cracks inside the particle cluster, connecting adjacent particle clusters, and finally leading to macroscopic failure^25^.

## Conclusion

In this work, we prepared Ti-based composites with in-situ TiC reinforcement. Firstly, graphite powder sheets were prepared as carbon source and the pyrolysis product of PVA reacts with matrix Ti to form TiC. By adding graphite powder sheet with different C content and studying the influence of microstructure evolution and mechanical properties of composite materials, we draw the following conclusions:

1. All composites are composed of only TiC and α-Ti and show uniform microstructure. When the carbon content is low, TiC presents approximately equiaxed rods; with the increase of C content, regular arrangement of discontinuous TiC particles reinforced TMCs were obtained. When the C content increased to 3.89wt%, a composite with multilayer microstructure was obtained.
2. The mechanical properties of Ti-based composites are significantly improved. As the C content increases, the ductility of the composites decreases. Both TMC-1 and TMC-2 composites achieve a good combination of strength and ductility, with UTS of 561.4 and 575.4 MPa, YS of 275 and 354 MPa, and EL of 8.8% and 6.4%, respectively.
3. The failure of all composites is triggered by TiC fracture. The discontinuous TiC structure can prevent the propagation of cracks, so that the composite can maintain good ductility, compared with the composite of multi-layer TiC structure.

## Data Availability

The datasets generated and analyzed during the current study are available from the corresponding author on reasonable request.
